# Evaluation of an enhanced ResNet-18 classification model for rapid On-site diagnosis in respiratory cytology

**DOI:** 10.1186/s12885-024-13402-3

**Published:** 2025-01-03

**Authors:** Wei Gong, Deep K. Vaishnani, Xuan-Chen Jin, Jing Zeng, Wei Chen, Huixia Huang, Yu-Qing Zhou, Khaing Wut Yi Hla, Chen Geng, Jun Ma

**Affiliations:** 1 Department of Pathology, Lishui Municipal Central Hospital, Lishui, 323000 Zhejiang Province China; 2 School of International Studies, Wenzhou Medical University, Ouhai District, Chashan, Wenzhou, 325035 Zhejiang Province China; 3 School of Clinical Medicine, Wenzhou Medical University, Ouhai District, Chashan, Wenzhou, Zhejiang Province, 325035 China; 4 Renji College, Wenzhou Medical University, Wenzhou, 325035 Zhejiang PR China; 5 Department of Archives, Lishui Second People’s Hospital, Liandu District, Lishui City, 323000 Zhejiang Province China; 6 Suzhou Institute of Biomedical Engineering and Technology, Chinese Academy of Sciences, Suzhou, 215163 Jiangsu Province China; 7 Department of Pathology, The First Affiliated Hospital of Wenzhou Medical University, Wenzhou, 325000 Zhejiang China

**Keywords:** Artificial Intelligence (AI), Lung Cancer, Computer-aided diagnosis, ResNet-18, Rapid on-site evaluation (ROSE)

## Abstract

**Objective:**

Rapid on-site evaluation (ROSE) of respiratory cytology specimens is a critical technique for accurate and timely diagnosis of lung cancer. However, in China, limited familiarity with the Diff-Quik staining method and a shortage of trained cytopathologists hamper utilization of ROSE. Therefore, developing an improved deep learning model to assist clinicians in promptly and accurately evaluating Diff-Quik stained cytology samples during ROSE has important clinical value.

**Methods:**

Retrospectively, 116 digital images of Diff-Quik stained cytology samples were obtained from whole slide scans. These included 6 diagnostic categories - carcinoid, normal cells, adenocarcinoma, squamous cell carcinoma, non-small cell carcinoma, and small cell carcinoma. All malignant diagnoses were confirmed by histopathology and immunohistochemistry. The test image set was presented to 3 cytopathologists from different hospitals with varying levels of experience, as well as an artificial intelligence system, as single-choice questions.

**Results:**

The diagnostic accuracy of the cytopathologists correlated with their years of practice and hospital setting. The AI model demonstrated proficiency comparable to the humans. Importantly, all combinations of AI assistance and human cytopathologist increased diagnostic efficiency to varying degrees.

**Conclusions:**

This deep learning model shows promising capability as an aid for on-site diagnosis of respiratory cytology samples. However, human expertise remains essential to the diagnostic process.

**Supplementary Information:**

The online version contains supplementary material available at 10.1186/s12885-024-13402-3.

## Background

Lung cancer currently has the second highest incidence rate and the highest cancer-related death rate globally. In China, it ranks first for both. In 2020 alone, there were 820,000 new lung cancer cases and 720,000 lung cancer-related deaths in China. Thus, lung cancer poses the greatest threat to human health and life, while also imposing an immense economic burden on patients [1, 2]. The diagnosis and staging of lung cancer increasingly rely on minimally invasive interventional procedures, including imaging-guided lung mass biopsies, bronchoscopic brush biopsies, bronchial biopsies, and lung peripheral nodule sampling biopsies under magnetic levitation navigation. Given the rapid development of personalized lung cancer treatments, pathologic typing and molecular pathology results are essential to guide therapeutic decisions. Consequently, obtaining adequate and representative specimens on-site is of utmost clinical importance, as it can reduce unnecessary repeat procedures and expenses, thereby conserving medical resources.

Currently, on-site judgments primarily utilize Diff-Quik staining. Compared to traditional Hematoxylin and Eosin (HE) staining and Papanicolaou staining, the Diff-Quik process significantly shortens staining time but provides less nuclear detail. Most domestic pathologists are unfamiliar with this technique and require specialized training to gain proficiency. Additionally, full-time cytologists, whether domestic or international, typically have high workloads, and there is a shortage of qualified personnel [3–5]. As a result, at least 30 min is required for an expert to provide on-site judgment of a single case, which is both inefficient and costly. Some hospitals resort to quick training programs to enable interventional clinicians to screen films on-site or employ remote cytology consultations via the internet after scanning films [6–8]. However, the former leads to lower diagnostic accuracy, while the latter necessitates significant capital investment and consumes valuable time and resources from superior hospitals. With advancements in digital imaging and machine learning, artificial intelligence-assisted cytology shows promise for rapid on-site diagnoses while reducing human subjectivity and increasing repeatability.

ResNet-18 is a convolutional neural network with an 18-layer architecture, comprising 17 convolutional layers and one fully connected layer. It can be trained by inputting pre-processed images and then used to classify new, unknown images. ResNet-18 has been successfully applied in medical imaging diagnostics, including radiology, histopathology, and cytopathology [9–12], though it has not yet been used for on-site cytopathology of respiratory specimens. This study aimed to assess the performance of an improved ResNet-18 classification model as an aid in on-site determinations of respiratory cytopathology samples.

## Materials and methods

### Sample information

A total of 739 respiratory specimens with on-site diagnoses were collected at our hospital from January 2022 to March 2023. Positive cases included those with biopsy or cell block confirmation and immunohistochemical verification of diagnosis. The sample comprised 96 squamous cell carcinoma cases, 64 adenocarcinoma cases, 6 non-small cell carcinoma cases (which could not be definitively categorized as squamous cell carcinoma, adenocarcinoma, or small cell carcinoma by morphology and immunohistochemistry), 50 small cell carcinoma cases, 1 carcinoid case, and 20 normal cytology cases, where biopsy, clinical data, and imaging showed no malignancy. A MAGSCANNER KF-PRO-005 digital pathology slide scanner (Zhejiang Ningbo Jiangfeng Bioinformation Technology Co., Ltd.) scanned Diff-Quik stained slides at 200X magnification to generate whole slide images (WSIs), automatically focusing on the smears. Two senior cytologists then captured 400X magnification images with a 2048 × 1024 pixel resolution. In cases of disagreement, another chief physician arbitrated and selected consensus images. Generally, 3–20 + images were captured per case. Images were de-identified before model testing.

### Image selection and representation

To illustrate the diversity and diagnostic challenges of the dataset, we included representative images from each diagnostic category. These images were selected based on their quality and the presence of characteristic features relevant to the diagnosis. The selected images represent carcinoid, normal cells, adenocarcinoma, squamous cell carcinoma, non-small cell carcinoma, and small cell carcinoma. Each image was annotated with its corresponding diagnosis and used in the training and testing of the ResNet-18 classification model (Please refer to supplementary Data – Sample Information file).

### ResNet-18 classification model

The artificial intelligence ResNet-18 classification model used in this study was developed in collaboration with the Suzhou Research Institute of the Chinese Academy of Sciences and Lishui Municipal Central Hospital. The model employs a ResNet-18 network enhanced by convolutional block attention modules (CBAM) [13].

### Workflow for Rapid On-Site diagnosis

**Fig. 1:**
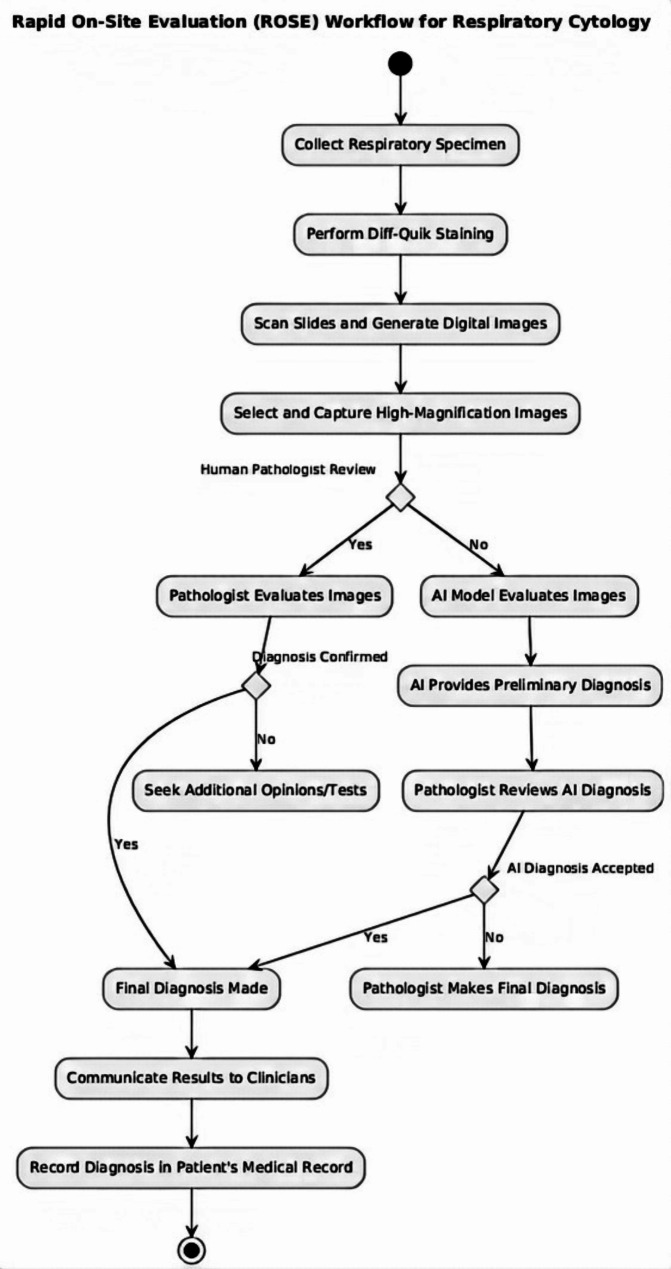
Workflow for Rapid On-Site Evaluation (ROSE) of respiratory cytology samples

To visualize the process of rapid on-site diagnosis in respiratory cytology, we developed a comprehensive workflow that outlines the steps from sample collection to diagnostic conclusion, incorporating both AI and pathologist evaluations. The workflow integrates deep learning models to assist pathologists in making real-time diagnostic decisions. The figure below presents the step-by-step procedure, demonstrating the clinical application of our diagnostic system (see Fig. 1).

### Model testing

Images were randomly selected from six categories: 8 images of carcinoid, 5 images of non-small cell carcinoma, 16 images of small cell carcinoma, 33 normal images, 20 images of squamous cell carcinoma, and 34 images of adenocarcinoma. These images were mixed and assigned random numbers. Three cytopathologists, each from a different hospital, along with an AI model, were tasked with diagnosing the individual images. The 116 test images were presented in a multiple-choice format, with the following answer options: precancerous, non-small cell carcinoma, small cell carcinoma, normal, squamous cell carcinoma, and adenocarcinoma. In cases of disagreement between the diagnoses of the physicians and the AI model, the correct diagnosis was selected as the final joint diagnosis. The three diagnosticians included a deputy chief physician with 28 years of experience, an attending physician with 16 years of experience, and a senior resident with 9 years of experience, all from different hospitals. Each of the physicians was qualified in cytological diagnosis.

### Combination of AI and cytopathologist diagnoses

In our study, we adopted a consensus approach to evaluate the combined diagnostic potential of AI and cytopathologists. Each of the 116 digital images was independently assessed by three cytopathologists and the AI model. Final diagnoses were determined based on consensus or specific criteria: a consensus diagnosis was recorded when all four entities agreed; if two or more cytopathologists agreed with the AI’s diagnosis, that was considered final; if the AI’s diagnosis aligned with the most experienced cytopathologist’s (deputy chief physician) diagnosis, it was deemed conclusive; and in cases of disagreement between the AI and cytopathologists, the diagnosis of the most experienced cytopathologist was selected to emphasize the value of human expertise in complex cases. This methodology is utilized for the combined diagnostic accuracy data with the full dataset provided as a supplementary file.

### Statistical methods

The following diagnostic performance metrics were calculated for each physician and the AI model based on the test data:

- **Sensitivity** = TP / (TP + FN) × 100%.
- **Specificity** = TN / (TN + FP) × 100%.
- **Positive Predictive Value (PPV)** = TP / (TP + FP) × 100%.
- **Negative Predictive Value (NPV)** = TN / (TN + FN) × 100%.
- **Accuracy** = (TP + TN) / (TP + FN + TN + FP) × 100%.

Where:

- **TP** = True Positive.
- **TN** = True Negative.
- **FP** = False Positive.
- **FN** = False Negative.

**Fig. 2:**
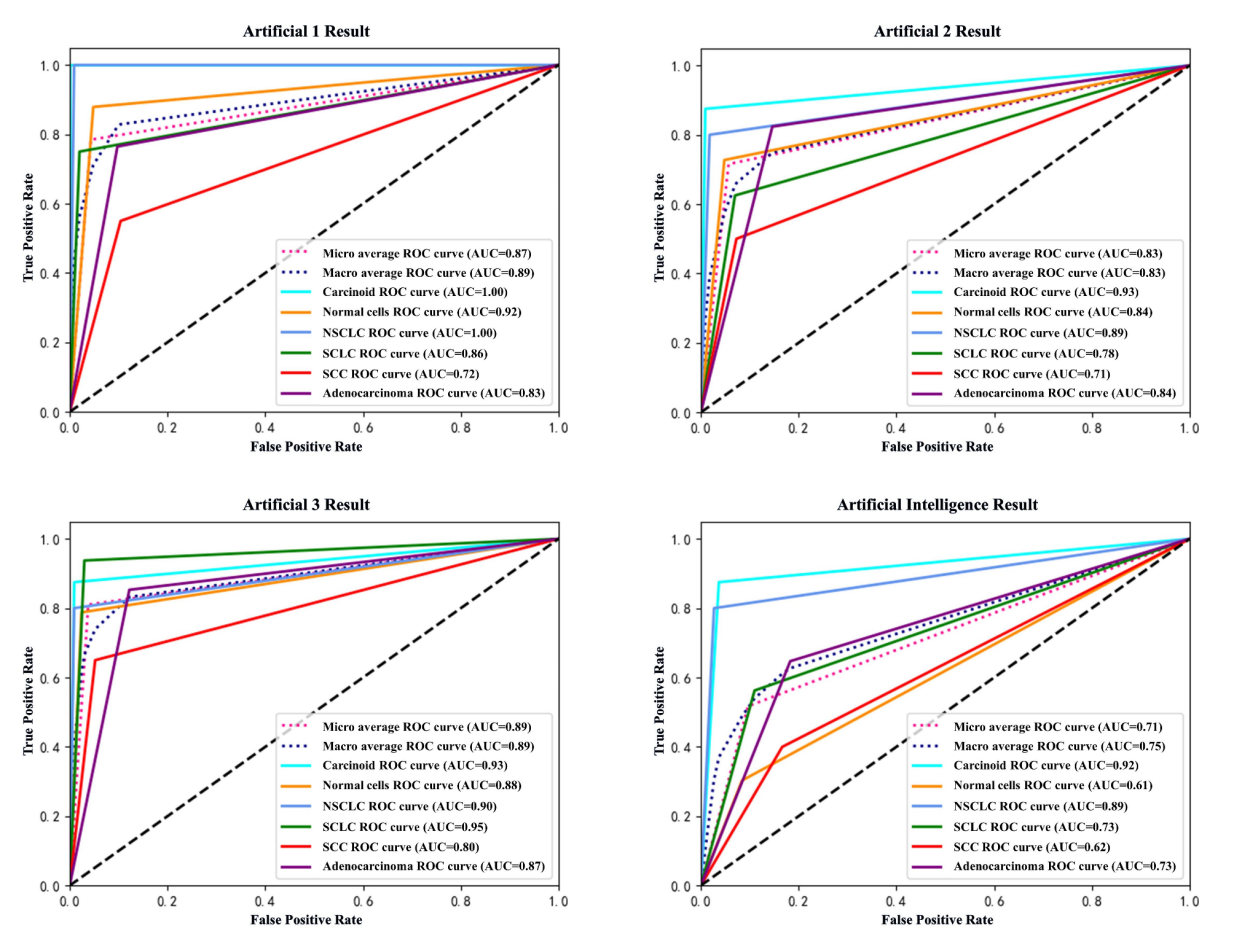
Multi-class ROC curves (macro-average methods)

Errors were counted as incorrect tumor type predictions or instances where a diagnosis could not be made. Multi-class classification metrics were calculated using the macro-average method, and multi-class ROC curves were generated in Python (Fig. 2).

## Results

Table 1 presents the diagnostic ability evaluation of the test for the three human doctors and the AI model. The four multi-class ROC curves provide a direct comparison of their respective diagnostic capabilities. Due to the experimental design, evaluating the diagnostic performance of human-AI joint diagnoses is challenging. However, in an ideal scenario where human and AI diagnoses differ, selecting the correct diagnosis would improve accuracy by 4.44%, 13.51%, and 11.79% for each physician, respectively (see Table 2).

**Table 1:** Statistical results of AI and human cytopathologist interpretation

	Sensitivity	Specificity	Positive Predictive Value	Negative Predictive Value	Accuracy
**P1%**	70.27	92.80	69.84	92.98	88.36
**P2%**	19.63	86.04	29.72	85.27	77 0.01
**P3%**	38.21	88.85	40.45	89.18	82.04
**AI%**	59.95	89.89	52.90	89.40	84.05

**Table 2:** Improved accuracy of AI-assisted human diagnosis

	Traditional Method Accuracy	Traditional + AI Accuracy	Difference
**P1%**	88.38	92.82	+ 4.44
**P2%**	77.01	90.52	+ 13.51
**P3%**	82.04	93.83	+ 11.79

**Fig. 3:**
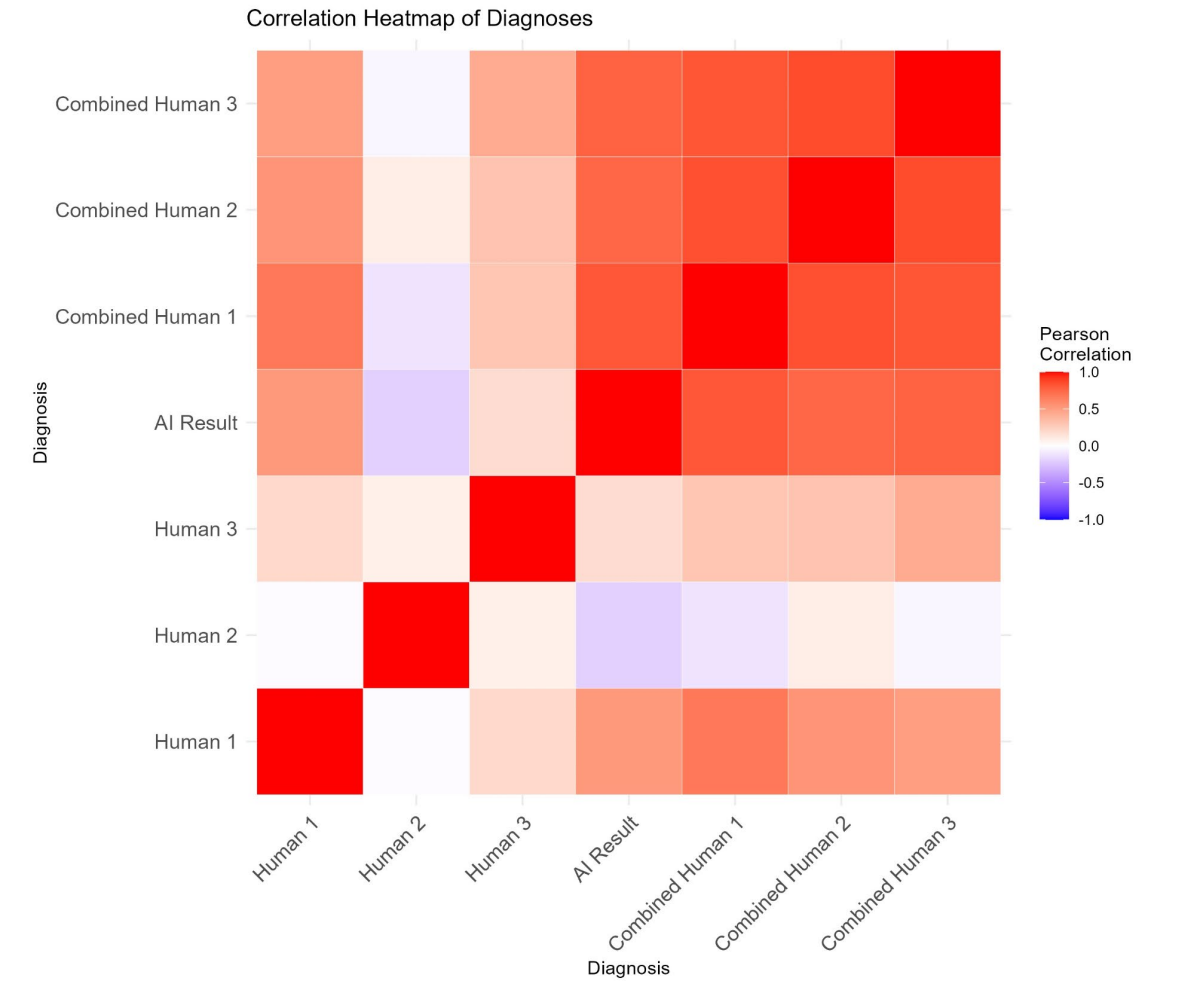
Pearson correlation heatmap of diagnoses

To further assess the agreement between the AI model and human experts, we computed Pearson correlation coefficients, which are visualized in the correlation heatmap (Fig. 3). This heatmap illustrates the correlation between the diagnostic outcomes of the AI model and the three human experts, as well as the combined results of the AI model and each human expert. The color gradient ranges from blue (negative correlation) to red (positive correlation), with white indicating no correlation. The heatmap reveals strong positive correlations across all comparisons, indicating a high level of agreement between the AI model and human experts in their diagnostic assessments.

The labels in the heatmap correspond to the following diagnostic methods:

- **Human 1**,** Human 2**,** Human 3**: Independent diagnostic assessments by three different human experts.
- **AI Result**: Diagnostic outcome provided by the AI model.
- **Combined Human 1**,** Combined Human 2**,** Combined Human 3**: Combined diagnostic outcomes of the AI model with each respective human expert.

The diagonal line from the top left to the bottom right of the heatmap, colored red, indicates perfect correlation, as each method is compared with itself. This figure underscores the consistency and reliability of the AI model in emulating the diagnostic capabilities of human experts.

The heatmap further highlights the strong positive correlations across all comparisons, suggesting a high level of diagnostic consistency between the AI model and the three physicians (P1, P2, P3). Combined results between the AI and each expert show particularly high agreement, underscoring the potential of joint diagnosis to improve overall accuracy. We observed that the AI’s diagnostic performance was comparable to that of human experts, with human accuracy being influenced by years of experience. Therefore, joint diagnosis proved to be significant.

(P1 is the deputy chief physician, P2 is the resident physician, and P3 is the attending physician).

## Discussion

The accuracy of cytological diagnosis is heavily influenced by the pathologist’s years of experience, accumulated expertise, and working conditions. It also depends on the adequacy of lesional cell quantity and quality, as well as proper slide preparation. After considering these confounding factors, the cytopathologist makes a holistic judgment based on background, cell arrangement, morphology, size, nuclear features (morphology, size, chromatin quality, nucleoli), nuclear membrane irregularity, N/C ratio, and cytoplasmic characteristics. These principles also apply to rapid on-site cytological diagnosis of respiratory specimens. In clinical practice, cytopathologists strive to differentiate tumor types, but poorly differentiated squamous cell carcinoma and adenocarcinoma often exhibit overlapping morphological features, necessitating immunohistochemical confirmation for definitive categorization. As a result, misclassification between these two types may occur.

The workflow outlined in this study was specifically designed for the rapid on-site diagnosis of lung cancer, focusing on subtypes such as squamous cell carcinoma, adenocarcinoma, and small cell carcinoma. However, we acknowledge that the principles behind this workflow—such as integrating AI-assisted cytological analysis, real-time diagnostics, and human expertise—could potentially be applied to other lung diseases, including interstitial lung diseases (ILD) and idiopathic pulmonary fibrosis (IPF). While these conditions are distinct from lung cancer, they present unique diagnostic challenges that could benefit from AI support.

It is important to note that the cytological features of ILD and IPF differ significantly from those of malignant lung tumors, requiring potential adaptations in the workflow for accurate diagnosis. At present, the workflow has not been validated for these diseases, and further research is needed to assess its applicability and diagnostic accuracy in non-cancerous conditions. Future studies could explore how the workflow might be adjusted for diseases like ILD or IPF, incorporating disease-specific features while maintaining the diagnostic efficiency of AI and human collaboration.

The suboptimal human diagnosis results in this study can primarily be attributed to the use of Diff-Quik staining for on-site diagnoses. This technique is unfamiliar to most cytopathologists in China, with few pathology labs routinely employing it. Compared to HE or Pap staining, Diff-Quik stains exhibit less nuclear detail and poorer cell cluster contrast, even with microscope adjustments. Thus, improving human on-site diagnosis requires specialized training and experience with this methodology. Additionally, the test provided only one image per question with six answer choices, a setup that differs from real-world conditions. The relatively low physician performance may not accurately reflect their capabilities in routine practice, as pathologists typically examine multiple slides and adjust focus when analyzing suspicious areas. Therefore, single-image testing cannot fully replicate physicians’ true diagnostic proficiency.

**Fig. 4:**
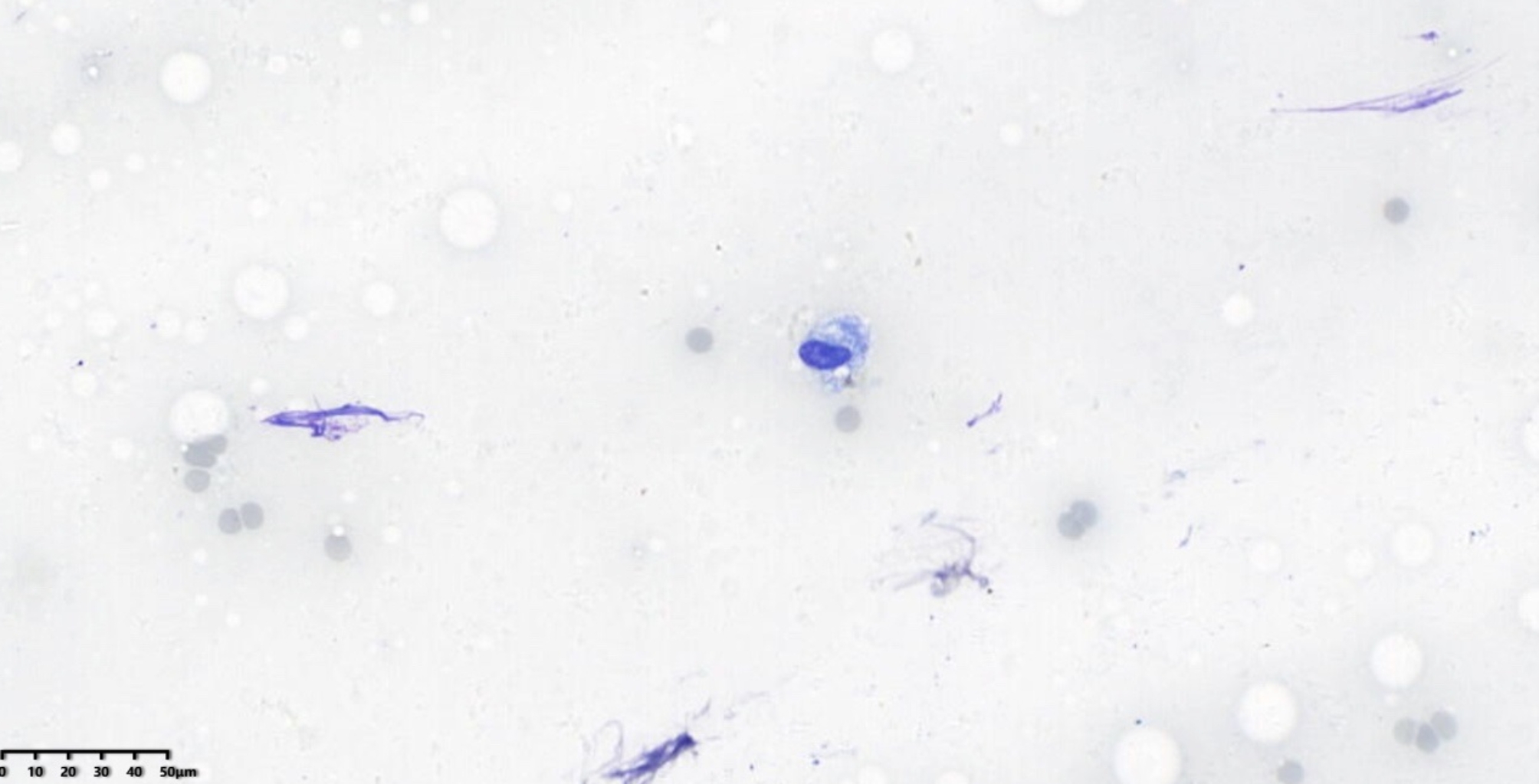
Adenocarcinoma, x400 magnification, Diff-Quik stain

**Fig. 5:**
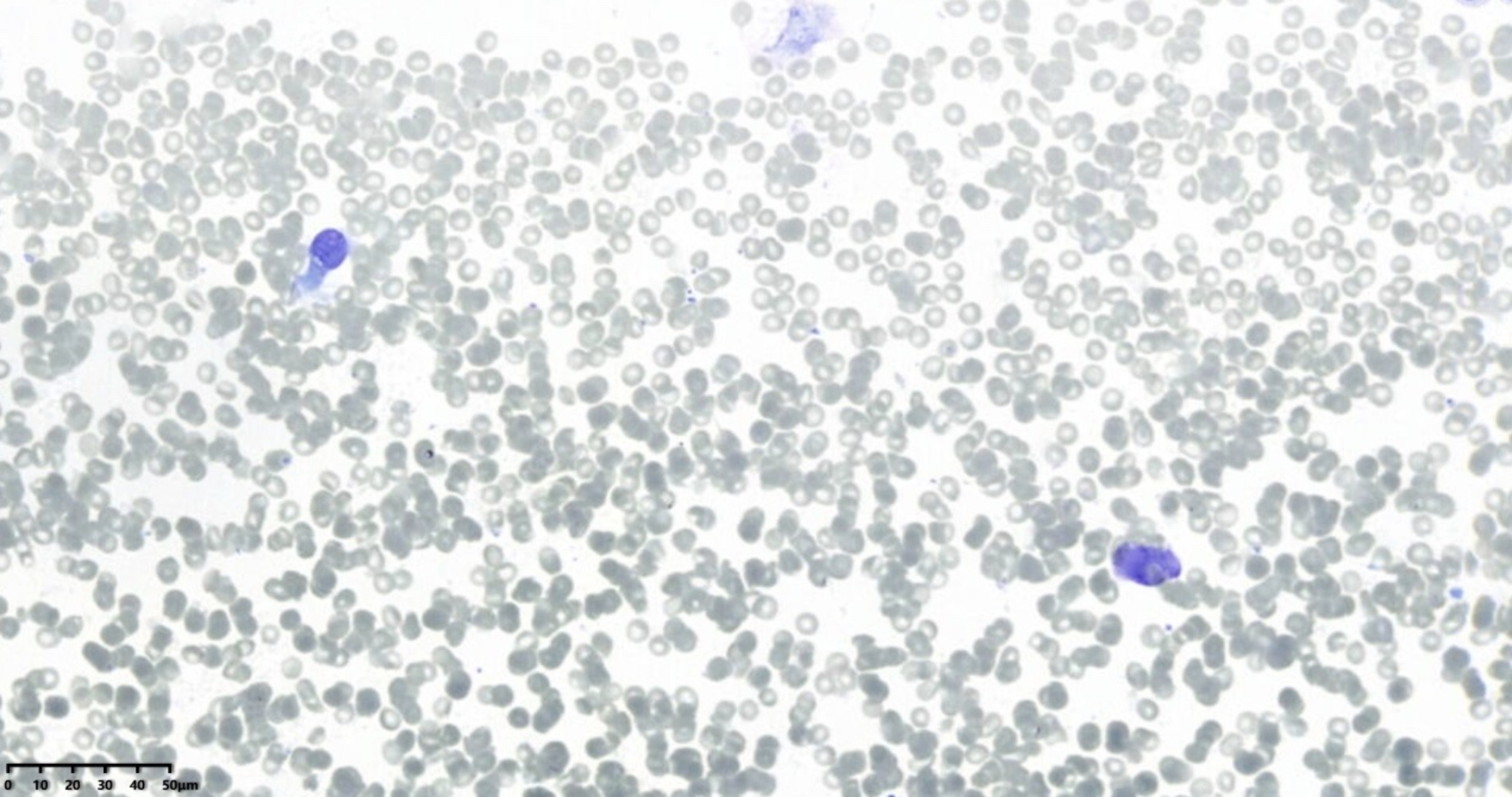
Small cell carcinoma, x400 magnification, Diff-Quik stain

ResNet is a deep convolutional neural network architecture originally designed for image classification, and it has been applied to histopathology and cytopathology. Its use for on-site diagnosis of respiratory cytology samples is novel. The improved ResNet-18 model performed exceptionally well in this study. As shown in Table 1, its diagnostic capability on images reached the level of human experts. Compared to human physicians, the AI model has the advantage of faster processing, taking only about 0.3 s to diagnose each image. Additionally, the AI’s performance is more objective and repeatable. A well-trained AI can detect subtle morphological changes that cytopathologists might overlook, given the considerable subjectivity and poorer reproducibility in human diagnoses (both intra- and inter-observer). For instance, in Fig. 4, an adenocarcinoma case was missed by all three pathologists because, without control cells as a background, they could not assess the size and shape of the cells properly, potentially overlooking enlarged nucleoli and thickened nuclear membranes. However, the AI identified the case as squamous cell carcinoma, which was closest to the gold standard. In Fig. 5, the AI correctly identified small cell carcinoma, while the human pathologists either erred or could not provide a definitive diagnosis. Retrospective analysis revealed normal bronchial epithelium in the upper left and a near-naked tumor cell in the lower right, which resembled normal bronchial cells and was easily overlooked by humans. Moreover, while humans can adjust the microscope focus to discern differences in morphology and arrangement, AI faces difficulties in diagnosing complex, high-density cell groups and accurately interpreting cellular arrangements. Advanced algorithms are needed to address these limitations [4, 14, 15].

**Fig. 6:**
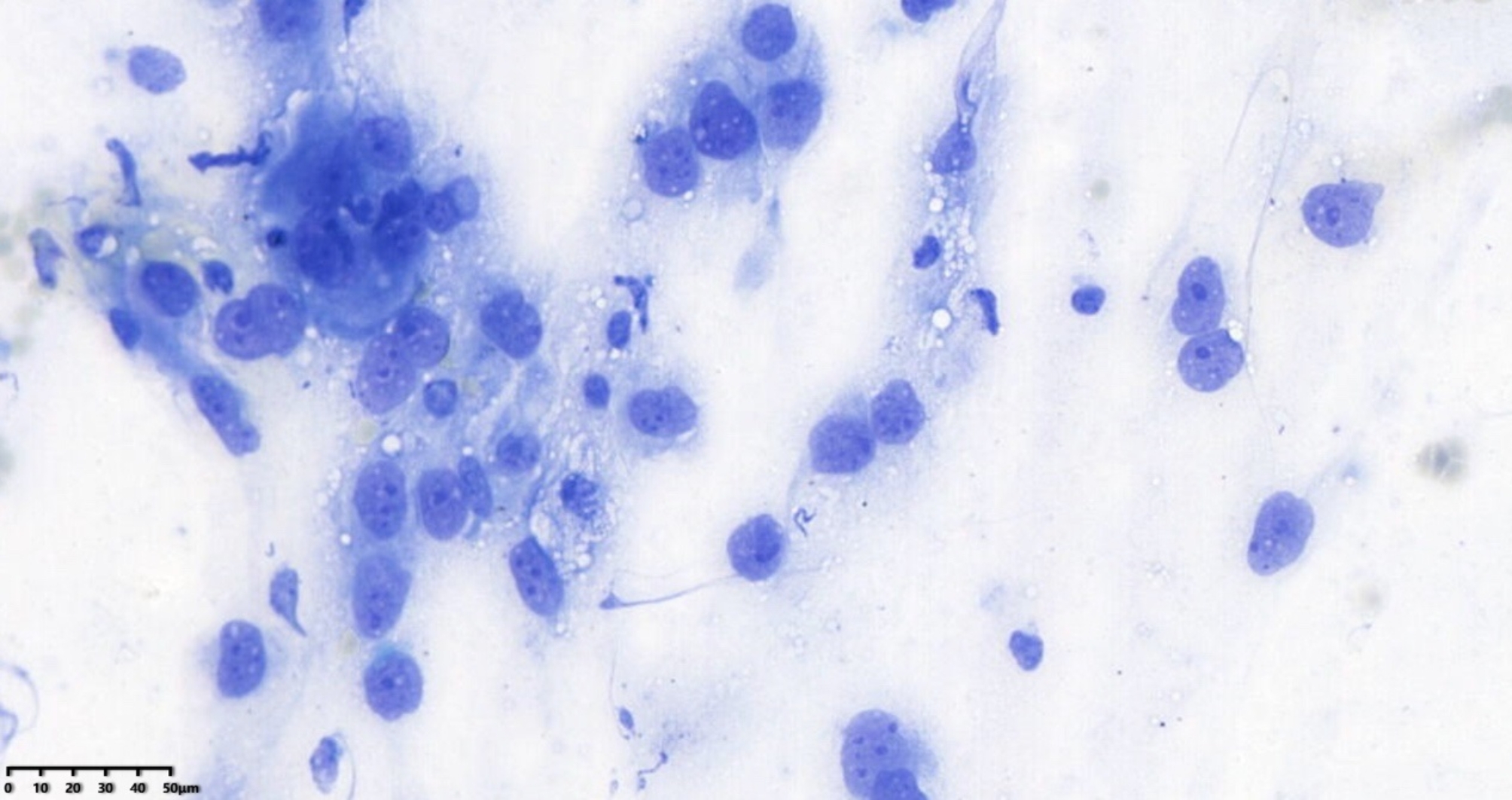
Non-small cell carcinoma, x400 magnification, Diff-Quik stain

Currently, AI algorithms in cytopathology are still in early development and cannot fully replace human expertise. They may make mistakes or overlook critical cytological features. For example, in Fig. 6, both the AI and one pathologist called the case normal, while the senior pathologist diagnosed it as non-small cell carcinoma. This discrepancy likely occurred because the cells resembled reactive epithelium, though their nuclear features differed from those of normal cells.

AI-assisted diagnosis, where the correct diagnosis is selected when human and AI diagnoses differ, has been shown to improve accuracy, as reflected in the literature [16]. However, human judgment remains essential in clinical practice. When discrepancies arise, professional knowledge and experience are required to assess whether the AI’s conclusion is correct and whether it should be adopted. Ultimately, humans must make the final cytological interpretation [17]. Fully relying on AI also introduces ethical, legal, and governance issues regarding its future application. Nonetheless, AI’s role in cytopathological diagnosis is likely to grow as machine learning and algorithms continue to evolve. Study limitations include using images from only one hospital. To evaluate real-world AI performance, multi-center participation and model training/validation on more cases are necessary to ensure the accuracy and reliability of the models. The ultimate goal is for AI to provide an integrative interpretation of entire digital slides, achieving diagnostic capabilities comparable to cytopathologists.

In our study, we used minimally invasive techniques such as fiber optic bronchoscopy, magnetic navigation bronchoscopy, and CT-guided transthoracic needle aspiration to obtain respiratory cytology samples. These methods are effective in providing diagnostic value while minimizing patient trauma. However, sampling bias, influenced by factors such as lesion location, heterogeneity, and operator experience, may impact the results. While efforts were made to ensure sample diversity and representativeness, the potential for bias remains a concern. Additionally, the morphological differences in tumor samples—such as the distinction between central necrotic areas and peripheral atypical squamous cells in squamous cell carcinoma or the comparison between marginally invasive cells and central mature tumor cells in adenocarcinoma—are significant but beyond the scope of this study. Furthermore, varying case exposure among cytopathologists at different hospital levels directly affects their diagnostic experience, with younger doctors more likely to consider preliminary diagnoses and more experienced doctors considering a wider range of differential diagnoses. Our deep learning model, based on ResNet-18, addresses this experience gap by assisting in diagnostic accuracy, demonstrating its potential to serve doctors across various hospital levels. Moving forward, we plan to expand our research to include a broader range of respiratory diseases, conduct multi-center studies, explore tumor heterogeneity, and optimize the model for better clinical application, ultimately enhancing AI’s role in cytological diagnosis and improving diagnostic services for patients.

While this study focuses on the Chinese context, particularly with the use of Diff-Quik staining, the findings have global relevance. In many resource-constrained settings, including both developing and developed countries, Diff-Quik staining offers a fast, cost-effective, and accessible alternative to more complex staining methods like HE or Pap. In regions where specialized training and advanced diagnostic tools are limited, this simple yet effective staining method can support timely and accurate on-site cytological diagnoses. Moreover, AI-assisted cytology, as demonstrated in this study, holds promise worldwide. While developed countries may have the infrastructure to adopt these technologies, AI can also be instrumental in improving diagnostic capabilities in developing countries, where access to trained pathologists may be limited. The integration of AI into cytopathology could reduce diagnostic variability and enhance the accuracy of diagnoses in diverse healthcare settings. As AI and staining techniques become more accessible globally, these methods could significantly improve diagnostic outcomes, particularly in areas with limited healthcare resources.

## Conclusion

In essence, AI and human cytopathologists form a dynamic partnership that amplifies each other’s strengths. By collaborating, they unlock the potential for improved diagnostic outcomes. AI lightens the workload and boosts diagnostic precision, while human expertise provides critical, high-level judgment, ensuring diagnoses of unmatched accuracy and reliability.

## Electronic supplementary material

Below is the link to the electronic supplementary material.

Supplementary Material 1

Supplementary Material 2

Supplementary Material 3

Supplementary Material 4

## Data Availability

The complete code for this research, including models, data preprocessing, training, testing, and evaluation, is available in the public GitHub repository (https://github.com/cir9/resnet-cbam-py).

## Data availability

All data generated and or analyzed during this study are included in this published article.

## Declarations

### Experimental protocol

The experimental protocols and procedures described in the study ‘Evaluation of an Enhanced ResNet-18 Classification Model for Rapid On-site Diagnosis in Respiratory Cytology’ were reviewed and approved by the Institutional Review Board and Ethics Committee of Lishui Municipal Central Hospital prior to initiation of the study. The approved protocols and procedures comply with ethical guidelines for human subjects’ research and ensure the privacy and safety of all study participants.

## Abbreviations

AI: Artificial intelligence
ROSE: Rapid on-site evaluation
DNA: Deoxyribonucleic acid
PCR: Polymerase chain reaction
HE: Hematoxylin and eosin
HDL: High-density lipoprotein
RNAi: RNA interference
WSIs: Whole slide images
N/C: Nuclear/cytoplasmic
TP: True positive
TN: True negative
FP: False positive
FN: False negative
CBAM: convolutional block attention module
ResNet-18: Residual Network 18
IHC: Immunohistochemistry
CT: Computed tomography
SC: Squamous cell carcinoma
AC: Adenocarcinoma
NSCLC: Non-small cell lung cancer
SCLC: Small cell lung cancer
AI-assisted CAD: Artificial intelligence-assisted computer-aided diagnosis
ILD: Interstitial lung disease
IPF: Idiopathic pulmonary fibrosis
NGS: Next-generation sequencing
FNA: Fine needle aspiration
AI-CAD: Artificial intelligence-computer-aided diagnosis
ROC: Receiver operating characteristic
P1: Pathologist 1 (deputy chief physician)
P2: Pathologist 2 (resident physician)
P3: Pathologist 3 (attending physician)
